# Supplementary material for: Realizing Blume-Capel Degrees of Freedom with Toroidal Moments in a Ruby Artificial Spin Ice
Source: ACS Nano. 2026 Jan 20;20(4):3423–34. doi: 10.1021/acsnano.5c13342 (PMC12874639; doi:10.1021/acsnano.5c13342)
Supplement: Supplementary file 1 [file nn5c13342_si_001.pdf]

Supporting Information:
Realizing Blume-Capel Degrees of Freedom with Toroidal Moments in a Ruby Artificial Spin Ice

Luca Berchialla[*,1,2], *Gavin M. Macauley*[*,1,2,†], *Flavien Museur*[*,1,2], *Tianyue Wang*[1,2], *Armin Kleibert*[3], *Peter M. Derlet*[1,4] *and Laura J. Heyderman*[*,1,2]

[1] Laboratory for Mesoscopic Systems, Department of Materials, ETH Zurich, 8093 Zurich, Switzerland

[2] PSI Center for Neutron and Muon Sciences, 5232 Villigen PSI, Switzerland

[3] Swiss Light Source, Paul Scherrer Institut, Villigen PSI 5232, Switzerland

[4] PSI Center for Scientific Computing, Theory and Data, 5232 Villigen PSI, Switzerland

† Present address: Department of Physics, Princeton University, Princeton, NJ 08540 USA.

* Email: l.berchialla@gmail.com, gm3291@princeton.edu, flavien.museur@psi.ch, laura.heyderman@psi.ch

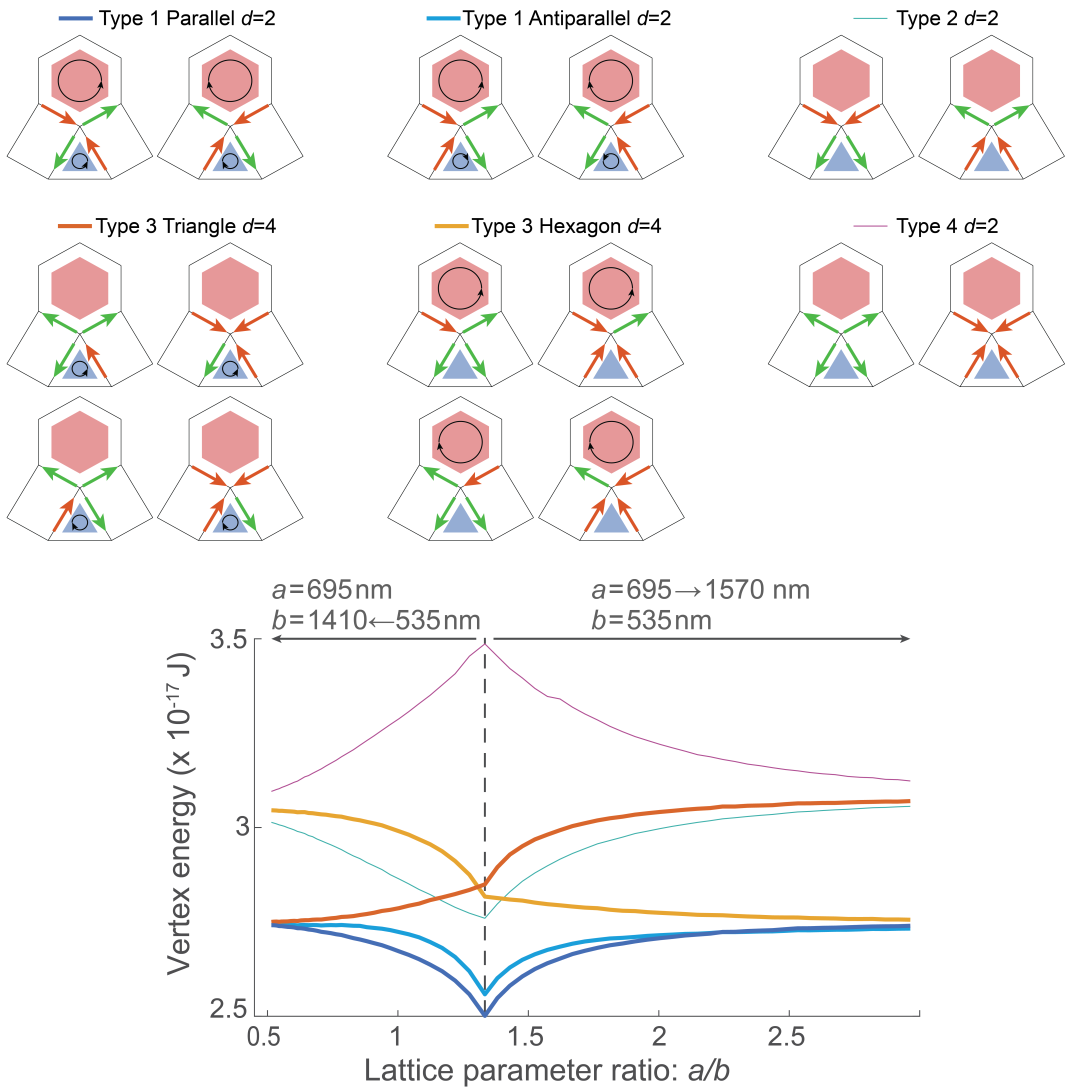

Figure S1. Vertex types and energies in the Ruby ASI. Upper Panel: all vertex configurations present in the Ruby ASI are grouped into six vertex types. The degeneracy *d* of each vertex type is indicated after the name of the vertex type. The vertex configurations are displayed over a schematic outline of the lattice (black lines). Within the schematic outline of the lattice, the hexagonal plaquettes are indicated by a red hexagon and the triangular plaquettes by a blue triangle. If all of the nanomagnets along the edge of a triangle or a hexagon are aligned head-to-tail, the sense of circulation is indicated by a black circular arrow. Lower Panel: vertex energies as a function of the lattice parameter ratio *a*/*b* obtained from micromagnetic simulations. The vertical dashed line corresponds to the lattice constant pair (a_{min}, b_{min}) = (695 nm, 535 nm). The graph has two parts as a function of *a*/*b*: starting from the vertical dashed line and moving to smaller *a*/*b* (i.e. leftwards from the dashed line), the lattice parameter *a* is kept constant at 695 nm while *b* is increased from 535 nm to 1410 nm; then, starting from the vertical dashed line and moving to larger *a*/*b* (i.e. rightwards from the dashed line), the lattice parameter *a* is increased from 695 nm to 1570 nm while *b* is kept constant at 535 nm.

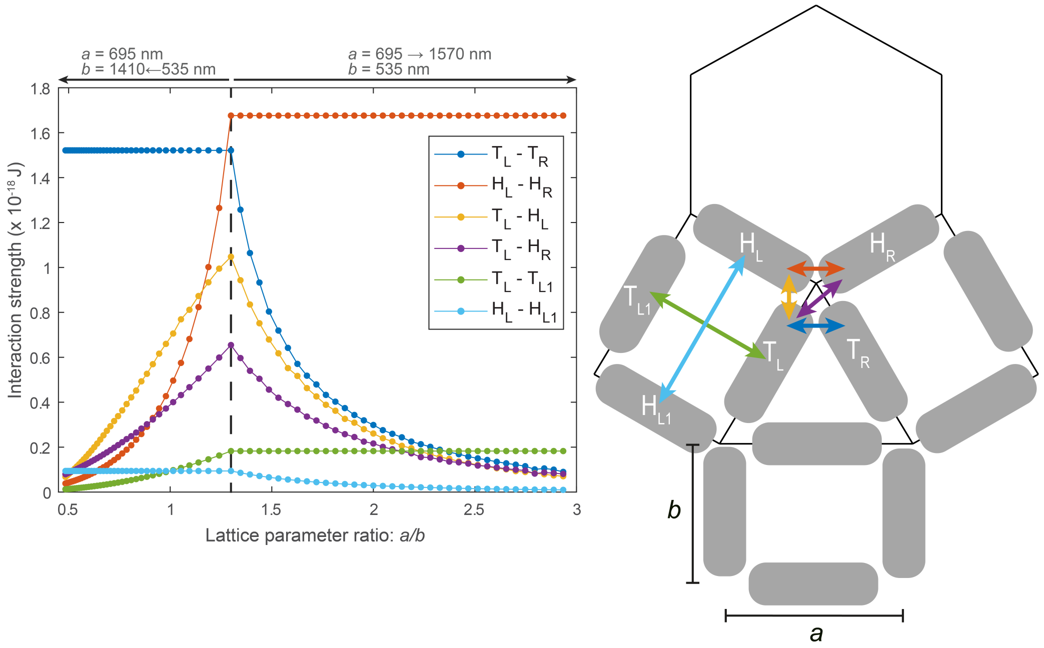

Figure S2. Pairwise interactions obtained from micromagnetic simulations. Pairwise interaction strengths as a function of the lattice parameter ratio a/b for all nanomagnet pairs within a unit cell. The vertical dashed line corresponds to the lattice constant pair (a_{min}, b_{min}) = (695 nm, 535 nm). The graph has two parts as a function of a/b: starting from the vertical dashed line and moving to smaller a/b (i.e. leftwards from the dashed line), the lattice parameter a is kept constant at 695 nm while b is increased from 535 nm to 1410 nm; then, starting from the vertical dashed line and moving to larger a/b (i.e. rightwards from the dashed line), the lattice parameter a is increased from 695 nm to 1570 nm while b is kept constant at 535 nm. The definitions of the nanomagnet pairs are shown on the right for an annotated unit cell of the Ruby ASI. We label nanomagnets T or H if they belong to a triangular or a hexagonal plaquette, respectively. The subscripts L and R denote whether a nanomagnet is on the left or the right with respect to the center of the unit cell, while the subscript 1, if present, indicates that the nanomagnet belongs to a different triangular or hexagonal plaquette than the one present in the unit cell schematic. In the main text, the strength of the interaction between nanomagnets T_L and T_R is called I_{Tr}, while the strength of the interaction between nanomagnets H_L and H_R is called I_{Hex}. The lattice parameters a and b are indicated.

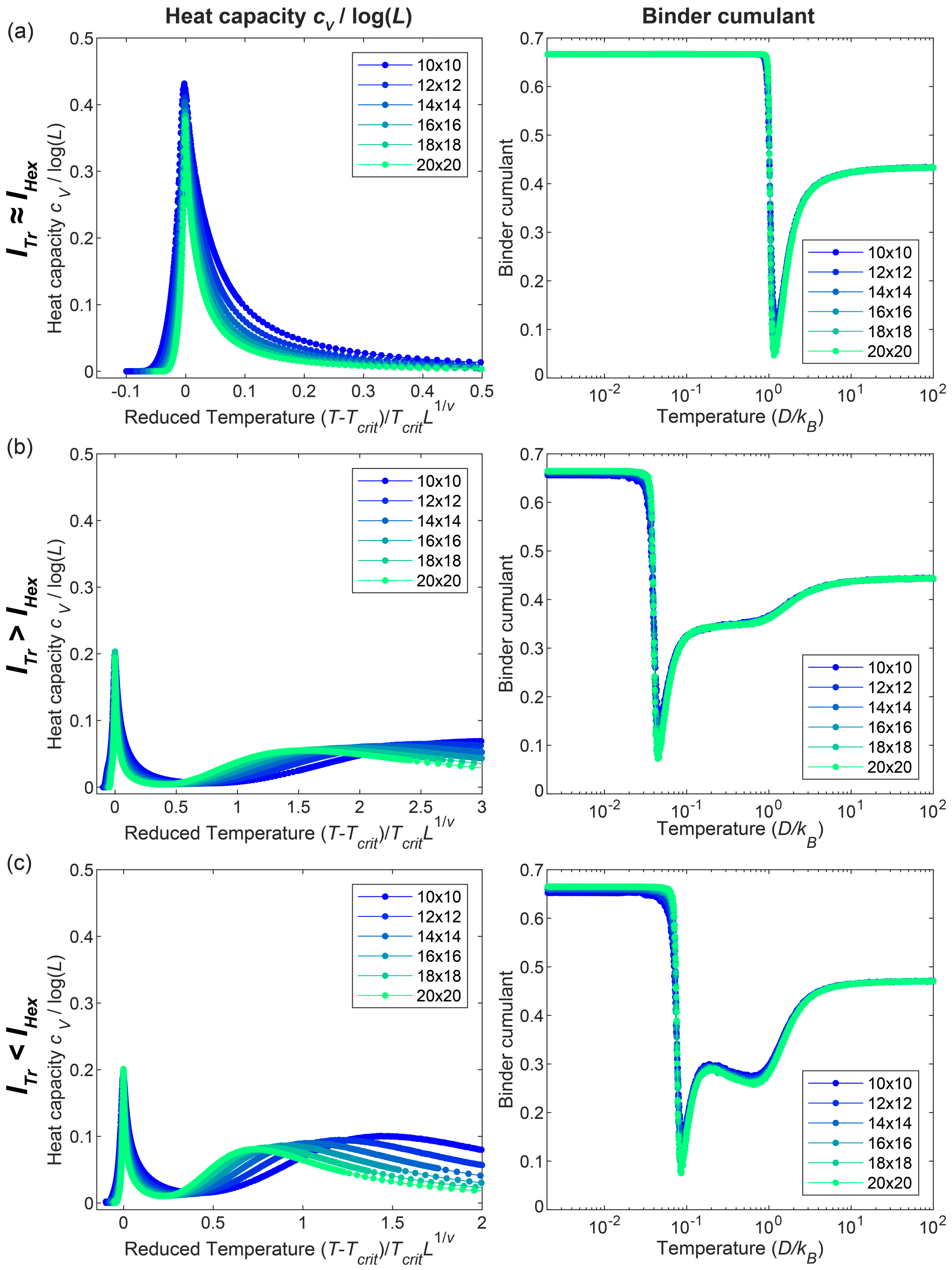

Figure S3. Finite-size scaling behavior of the Ruby ASI as obtained from Monte Carlo simulations. On the left, the scaled specific heat capacity, c_V / log(L), is plotted against the reduced temperature, which has been obtained from the Binder 4th order cumulant analysis. The curves are for a range of systems sizes L going from 10 ×10 to 20 × 20 unit cells. The curves appear to collapse when assuming the relevant critical exponent for a 2D Ising transition, namely ν = 1. On the right, the Binder cumulant is plotted as a function of temperature. The finite size scaling analysis has been performed on the three cases of $I_{Tr} \approx I_{Hex}$ (a = 695 nm, b = 535 nm) in panel (a), $I_{Tr} > I_{Hex}$ (a = 695 nm, b = 1410 nm) in panel (b) and $I_{Tr} < I_{Hex}$ (a = 1570 nm, b = 535 nm) in panel (c).

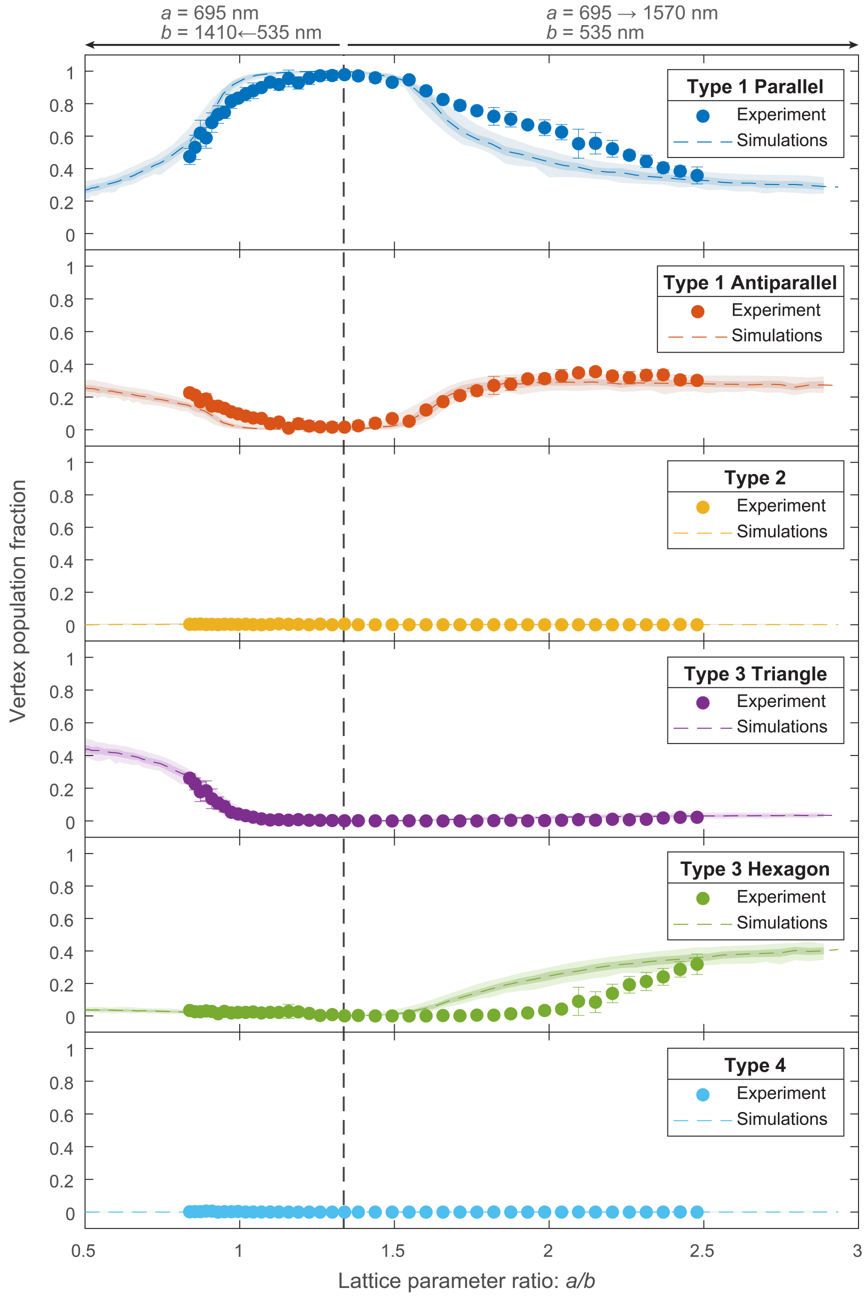

Figure S4. Vertex populations in as-grown configurations given as fractions of the total population as a function of the lattice parameter ratio. For all the graphs, each data point is the average of the represented quantity as extracted from MFM measurements of four identical Ruby ASIs, and the error bars indicate the standard deviation of as-grown samples for each lattice parameter. The dashed line indicates average values obtained from 100 individual Monte Carlo simulations at a temperature $k_B T/D \approx 0.469$. The inner colored area indicates the standard deviation while the outer lightly colored area indicates the maximum and minimum values. The vertical dashed line corresponds to the lattice constant pair $(a_{\min}, b_{\min}) = (695\text{ nm}, 535\text{ nm})$. The graph has two parts as a function of a/b: starting from the vertical dashed line and moving to smaller a/b (i.e. leftwards from the dashed line), the lattice parameter a is kept constant at 695 nm while b is increased from 535 nm to 1410 nm; then, starting from the vertical dashed line and moving to larger a/b (i.e. rightwards from the dashed line), the lattice parameter a is increased from 695 nm to 1570 nm while b is kept constant at 535 nm.

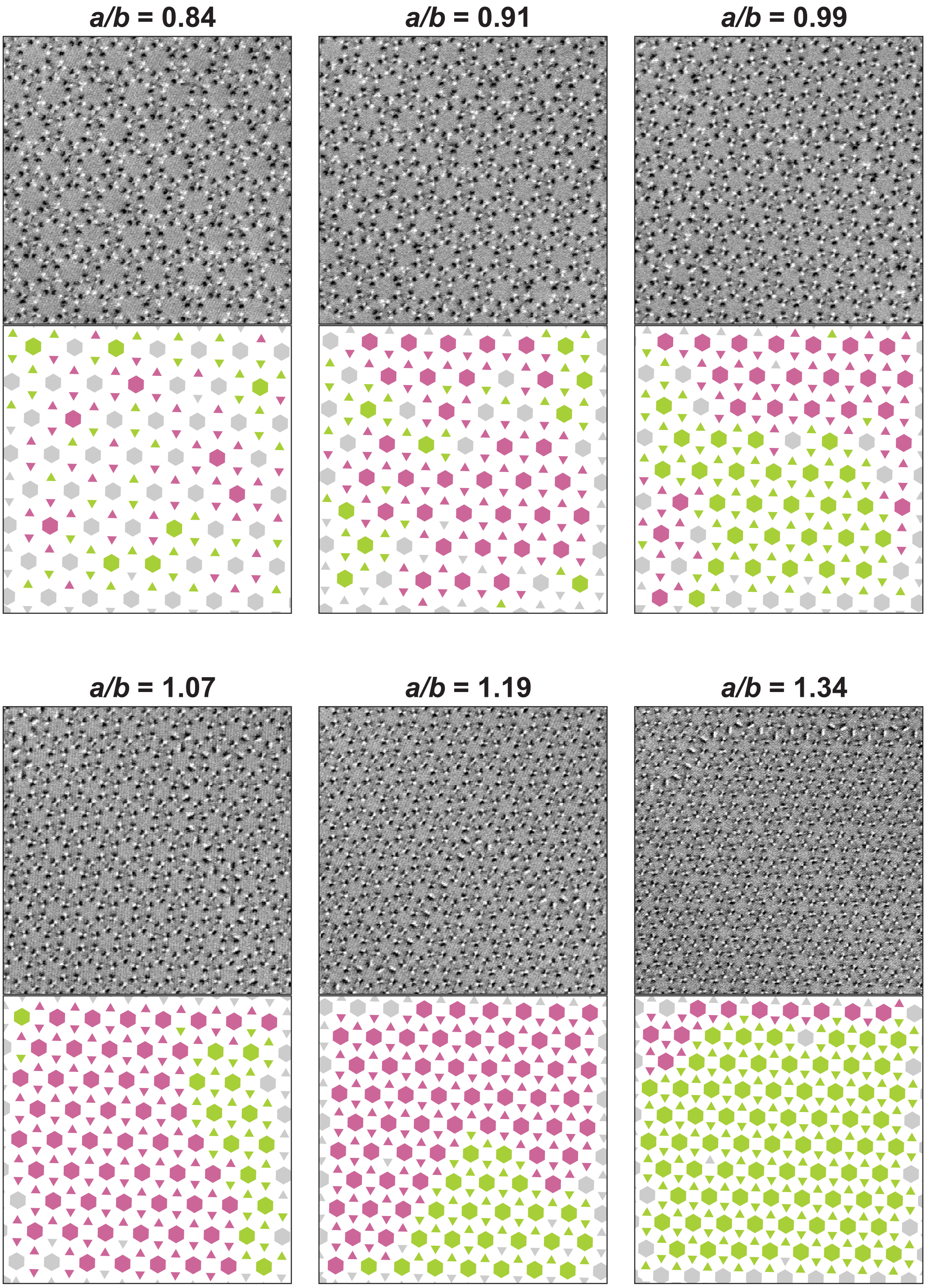

Figure S5. Ruby ASI as-grown configurations for increasing *a/b* – part I. For selected *a/b*, from 0.84 up to 1.34, MFM images and toroidal moment maps of as-grown configurations are given. Pink triangular and hexagonal plaquettes represent positive fully formed toroidal moments. Green triangular and hexagonal plaquettes represent negative fully formed toroidal moments. Gray triangular and hexagonal plaquettes indicate plaquettes that do not have a fully formed toroidal moment or, equivalently, where not all of the macrospins point head-to-tail.

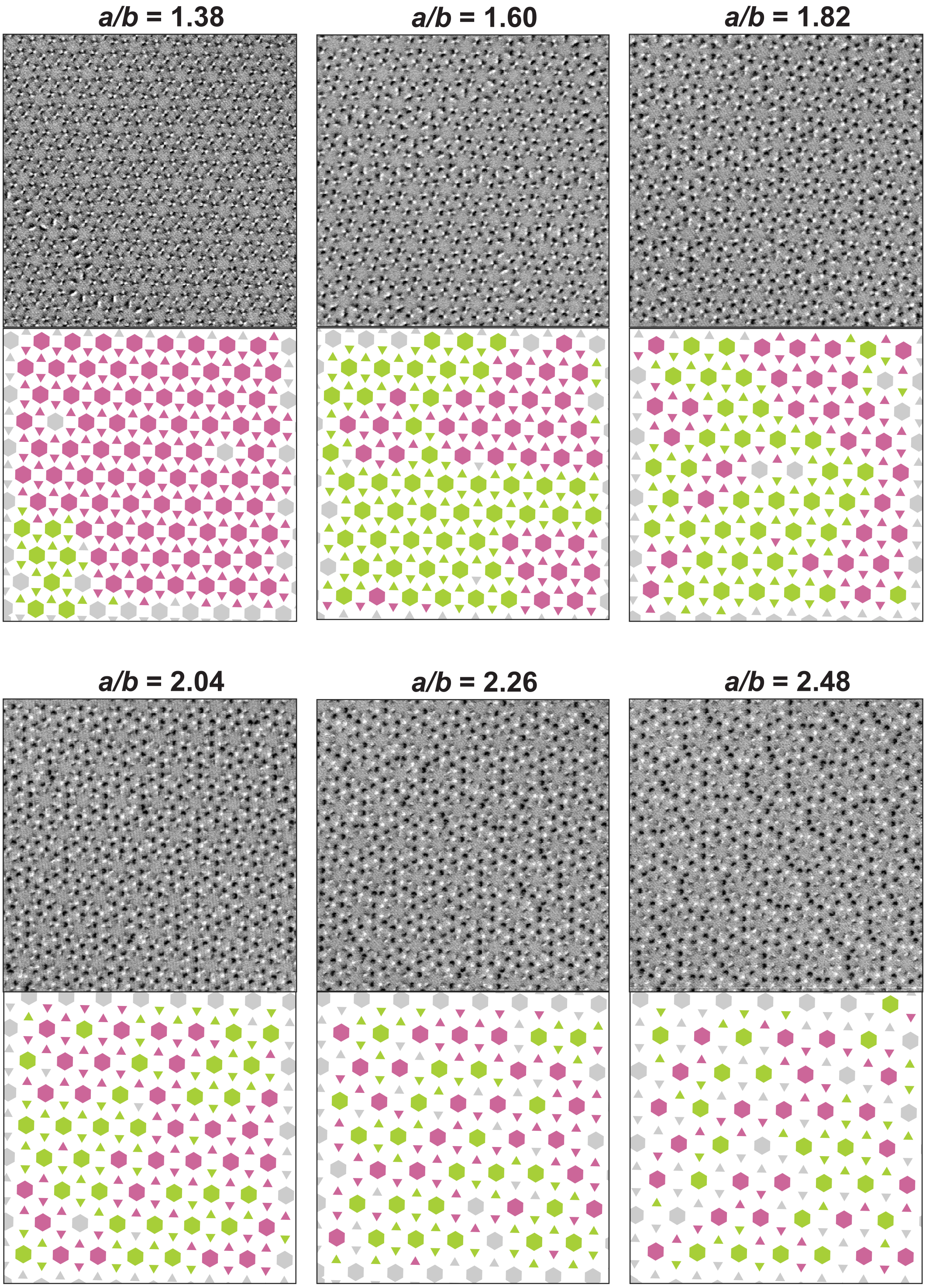

Figure S6. Ruby ASI as-grown configurations for increasing *a/b* – part II. For selected *a/b*, from 1.38 up to 2.48, MFM images and toroidal moment maps of as-grown configurations are given. Pink triangular and hexagonal plaquettes represent positive fully formed toroidal moments. Green triangular and hexagonal plaquettes represent negative fully formed toroidal moments. Gray triangular and hexagonal plaquettes indicate plaquettes that do not have a fully formed toroidal moment or, equivalently, where not all of the macrospins point head-to-tail.

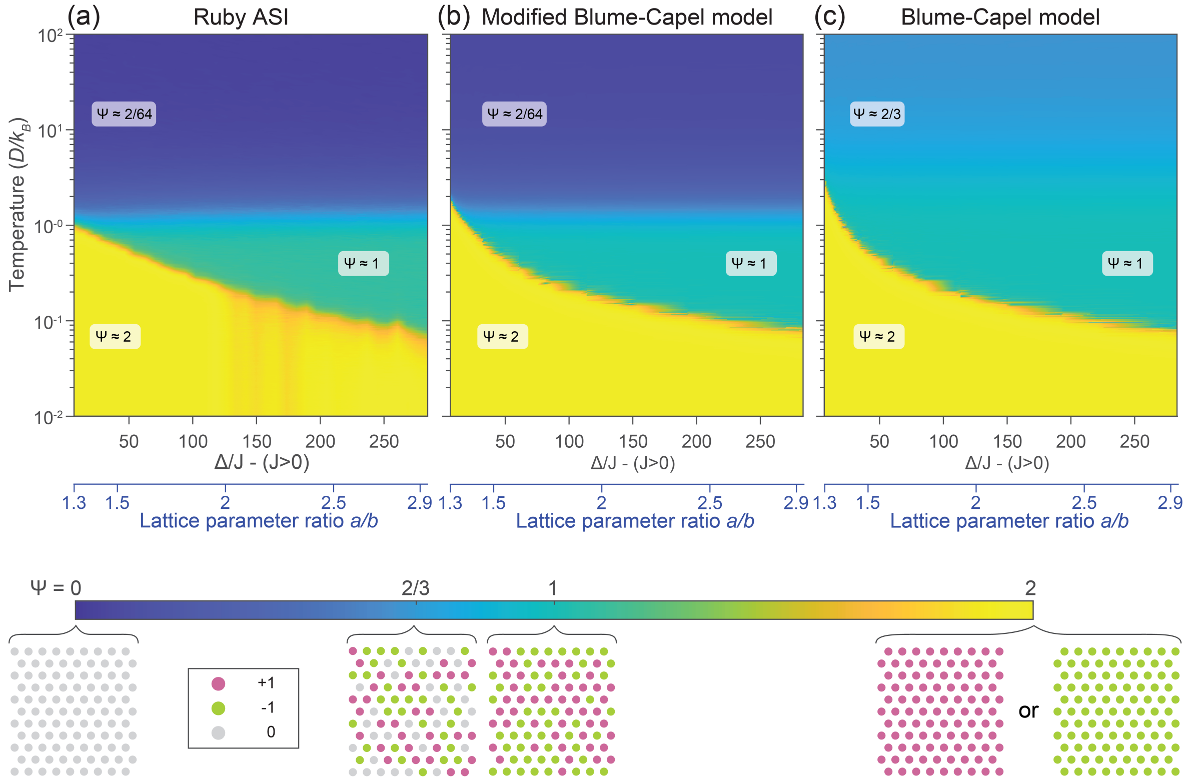

Figure S7. Blume-Capel model for hexagonal toroidal moments on a triangular lattice. Color plots of the figure of merit $\Psi = (n_{+1} + n_{-1}) + |n_{+1} - n_{-1}|$ as function of temperature and Δ/J for: (a) the Ruby ASI, where the full spin ensemble is considered and coupled through point-dipolar interactions; (b) the modified Blume-Capel model; and (c) the Blume-Capel model. The Monte Carlo simulations of the Blume-Capel models are performed in terms of Δ/J, which is dictated by the lattice parameter ratio a/b. The quantities n_{+1} and n_{-1} are the fractional populations of fully formed toroidal moments in the $t = +1$ and $t = -1$ states, respectively. In (a) and (b), $\Psi \approx 2/64$ at high temperature (dark blue region) since there are few fully formed toroidal moments, while in (c), $\Psi \approx 2/3$ at high temperature (light blue region) since the toroidal moments are generated to be 0, $+1$ or -1 with equal probabilities. In all of the models, over an intermediate temperature range, the system exists in the paratoroidic phase, with $\Psi \approx 1$ (green region), where all toroidal moments are fully formed but remain uncorrelated with each other, adopting one of the two allowed values, ± 1, at random. Similarly, the ground state of all of the models is the ferrotoroidic phase, with $\Psi = 2$ (yellow region), where all of the toroidal moments are in the same state, i.e. all have $t = +1$ or all have $t = -1$.

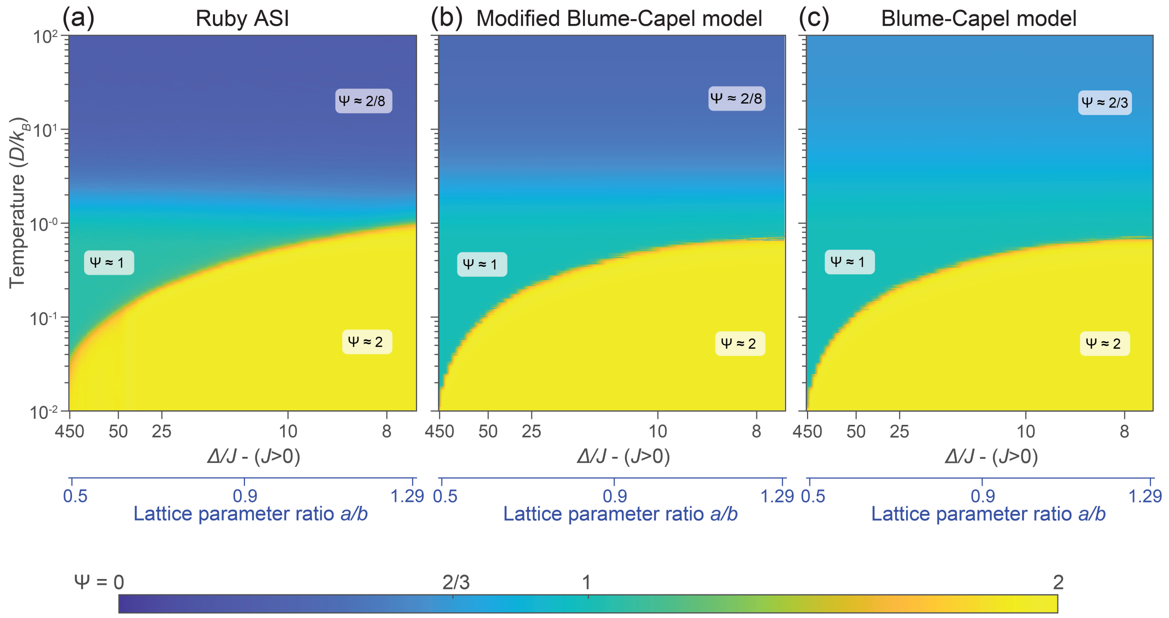

Figure S8. Blume-Capel model for triangular toroidal moments on a hexagonal lattice. Color plots of the figure of merit $\Psi = (n_{+1} + n_{-1}) + |n_{+1} - n_{-1}|$ as a function of temperature and Δ/J for: (a) the Ruby ASI, where the full spin ensemble is considered and coupled through point-dipolar interactions; (b) the modified Blume-Capel model; and (c) the Blume-Capel model. The Monte Carlo simulations of the Blume-Capel models are performed in terms of Δ/J, which is dictated by the lattice parameter ratio *a*/*b*. The quantities n_{+1} and n_{-1} are the fractional populations of fully formed toroidal moments in the $t = +1$ and $t = -1$ states, respectively. In (a) and (b), $\Psi \approx 2/8$ at high temperatures, giving a dark blue region, since there are only a few fully formed toroidal moments, while in (c) $\Psi \approx 2/3$ at high temperature, giving a light blue region, since the toroidal moments are 0, $+1$ or -1 with equal probabilities. In all of the models, over an intermediate temperature range, the system exists in the paratoroidic phase, with $\Psi \approx 1$ (green region), since all toroidal moments are fully formed but remain uncorrelated with each other, adopting one of the two allowed values, ± 1, at random. Similarly, in all of the models, the ground state is the ferrotoroidic phase with $\Psi = 2$ (yellow region), where all of the toroidal moments are in the same state, i.e. all have $t = +1$ or all have $t = -1$.

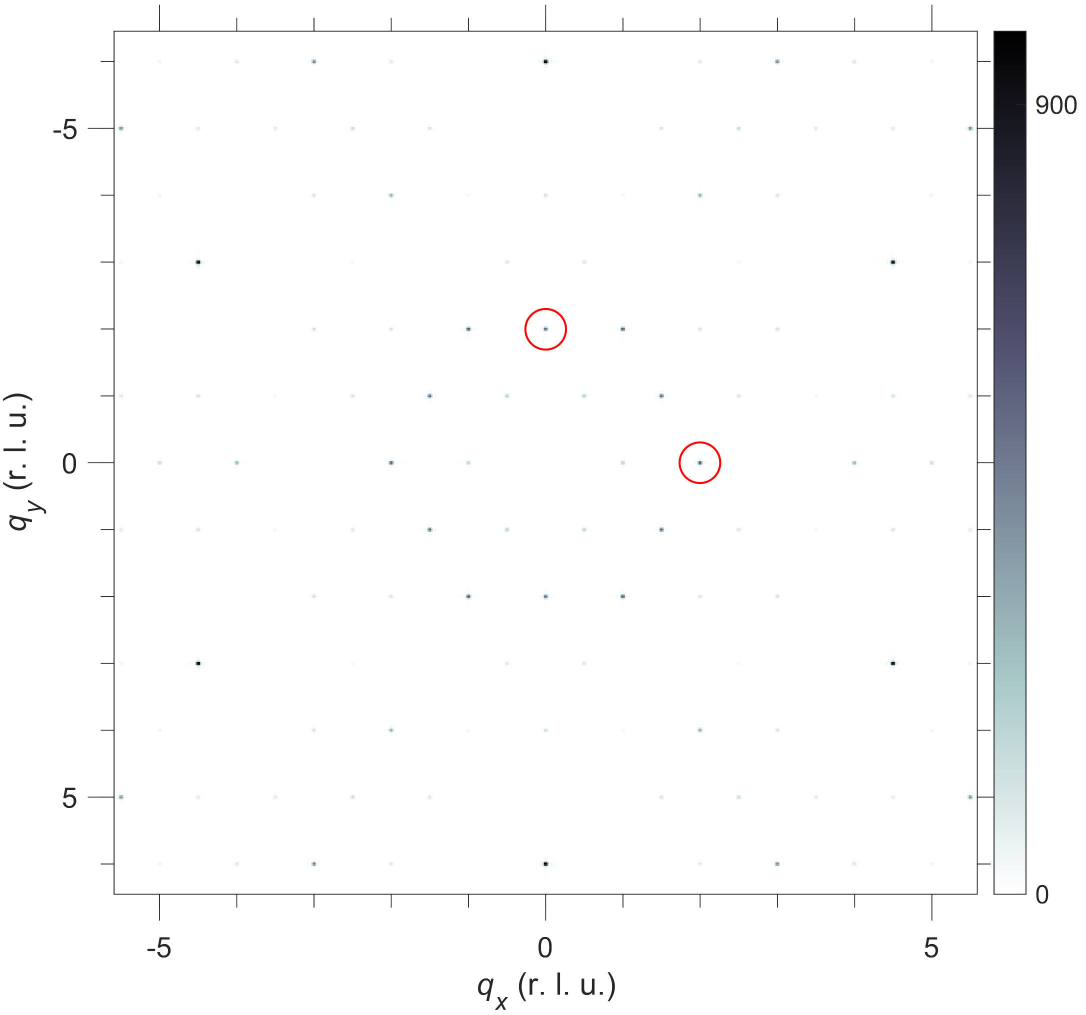

Figure S9. Magnetic structure factor averaged over 100 independent spin configurations obtained from the Monte Carlo simulations, taken at the effective temperature reached by thermal annealing in the experiment, for the case where $I_{\mathrm{Tr}} \approx I_{\mathrm{Hex}}$. Here, the original Bragg peaks are shown at their true scale in terms of number of pixels. This contrasts with the middle panel of Figure. 3c in the main text, where the peaks were artificially enlarged to ensure that they were visible. The red circles indicate Bragg peaks appearing at $Q_{xy} = \{\ [\ 4(a + b\sqrt{3}), 0\ ];\ [\ 0, 2(\sqrt{3}a + 3b)\ ]\ \}$.

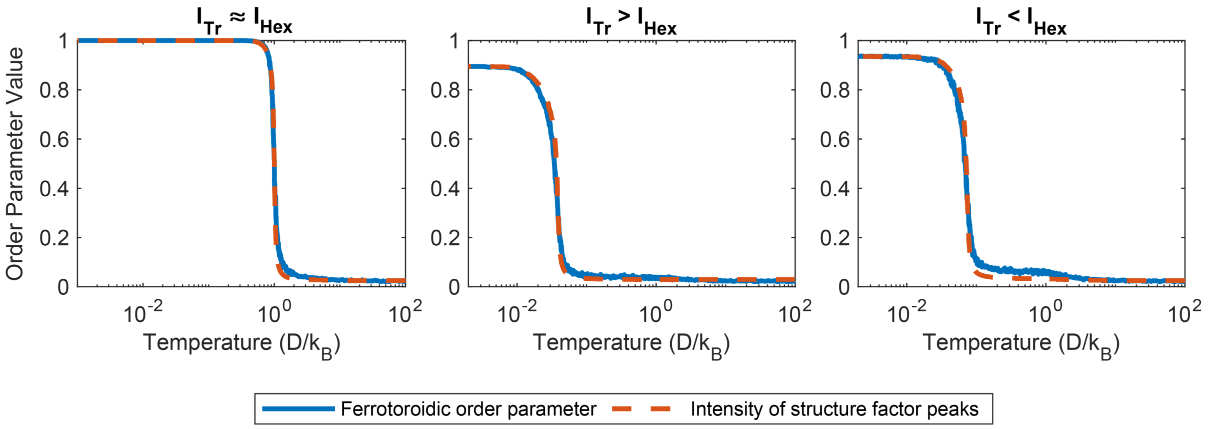

Figure S10. Temperature dependence of the absolute value of the ferrotoroidic order parameter $|\Phi|$ (solid blue line) and the sum of the intensity of the magnetic structure factor for the two points $Q_{xy} = \{\ [4(a + b\sqrt{3}),\ 0];\ [0, 2(\sqrt{3}a + 3b)]\ \}$ (dashed red line), which correspond to the Bragg peaks indicated with red circles in Supplementary Figure 9. The intensity of the structure factor peaks has been rescaled to match the ferrotoroidic order parameter at zero temperature.

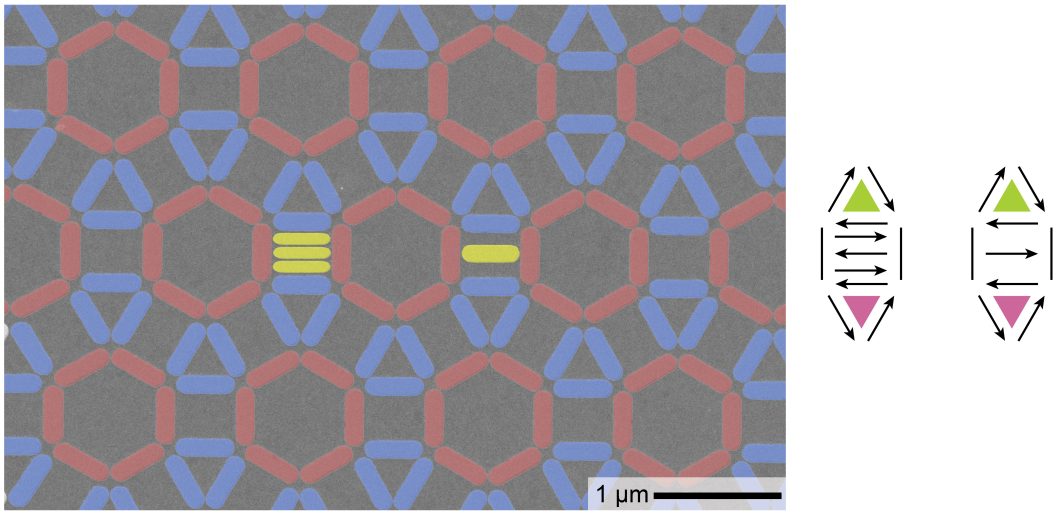

Figure S11. Colored scanning electron micrograph of the Ruby ASI with the highest density of nanomagnets, (a_{min}, b_{min}) = (695 nm, 535 nm). Nanomagnets lining hexagons (triangles) are colored in red (blue). An odd number of nanomagnets, shown in yellow, can be inserted to promote an effective antiferromagnetic coupling between toroidal moments associated with the triangular plaquettes. Two examples are given with one and three nanomagnets inserted, and the resulting macrospin orientation is given to the right.
